# Supplementary material for: Parental Autonomy Support and Mental Health among Chinese Adolescents and Emerging Adults: The Mediating Role of Self-Esteem
Source: Int J Environ Res Public Health. 2022 Oct 28;19(21):14029. doi: 10.3390/ijerph192114029 (PMC9653793; doi:10.3390/ijerph192114029)
Supplement: Supplementary file 1 [file ijerph-19-14029-s001.zip › ijerph-1958104-supplementary.pdf]

Supplementary Materials

Table S1

Results of measurement invariance of all scales between genders in Study 1

Model	χ^2/df	CFI	RMSEA	△CFI	△RMSEA
1.Parental autonomy support					
Configural Invariance	10.866	0.910	0.078		
Metric Invariance	10.036	0.910	0.075	0.000	0.003
Scalar Invariance	9.331	0.910	0.072	0.000	0.003
2. Self-esteem					
Configural Invariance	4.399	0.933	0.046		
Metric Invariance	4.275	0.926	0.045	0.007	0.001
Scalar Invariance	4.106	0.920	0.044	0.006	0.001
3. Life satisfaction					
Configural Invariance	4.460	0.988	0.046		

Metric Invariance	4.573	0.982	0.047	0.006	0.001
Scalar Invariance	3.946	0.978	0.043	0.004	0.004
4. Emotional symptoms					
Configural Invariance	1.552	0.996	0.018		
Metric Invariance	1.322	0.996	0.014	0.000	0.004
Scalar Invariance	3.417	0.963	0.039	0.033	0.025

Note. *N* = 1617.

Table S2

Results of measurement invariance of all scales between genders in Study 2

Model	χ^2 /df	CFI	RMSEA	△CFI	△RMSEA
1.Parental autonomy support					
Configural Invariance	7.954	0.924	0.074		
Metric Invariance	7.400	0.924	0.071	0.000	0.003
Scalar Invariance	7.088	0.922	0.069	0.002	0.002
2. Self-esteem					
Configural Invariance	8.810	0.942	0.078		
Metric Invariance	7.839	0.941	0.073	0.001	0.005
Scalar Invariance	7.383	0.938	0.071	0.003	0.002
3. Life satisfaction					
Configural Invariance	8.358	0.984	0.076		
Metric Invariance	6.357	0.984	0.065	0.000	0.011

Scalar Invariance	5.992	0.979	0.063	0.005	0.002
4. Depressive symptoms					
Configural Invariance	3.619	0.936	0.045		
Metric Invariance	3.538	0.935	0.045	0.001	0.000
Scalar Invariance	3.558	0.931	0.045	0.004	0.000

Note. N = 1274.
